# Supplementary material for: The impact of rearing environment on C. elegans: phenotypic, transcriptomic and intergenerational responses to 3D enriched habitats
Source: Biol Open. 2026 Feb 18;15(2):bio062282. doi: 10.1242/bio.062282 (PMC12958300; doi:10.1242/bio.062282)
Supplement: Supplementary information [file biolopen-15-062282-s1.pdf]

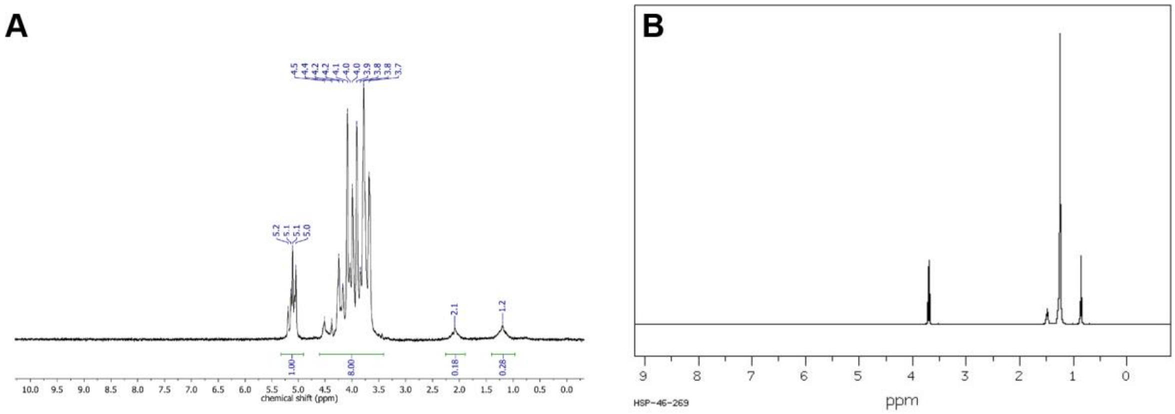

Fig. S1. ^{1}H HR-MAS NMR analysis confirms the absence of detergent residues. (A) Full spectral region of the decellularized apple scaffold. (B) Full spectral region of the decellularizing agent sodium dodecyl sulfate (SDS) (National Institute of Advanced Industrial Science and Technology, 1999)**.**

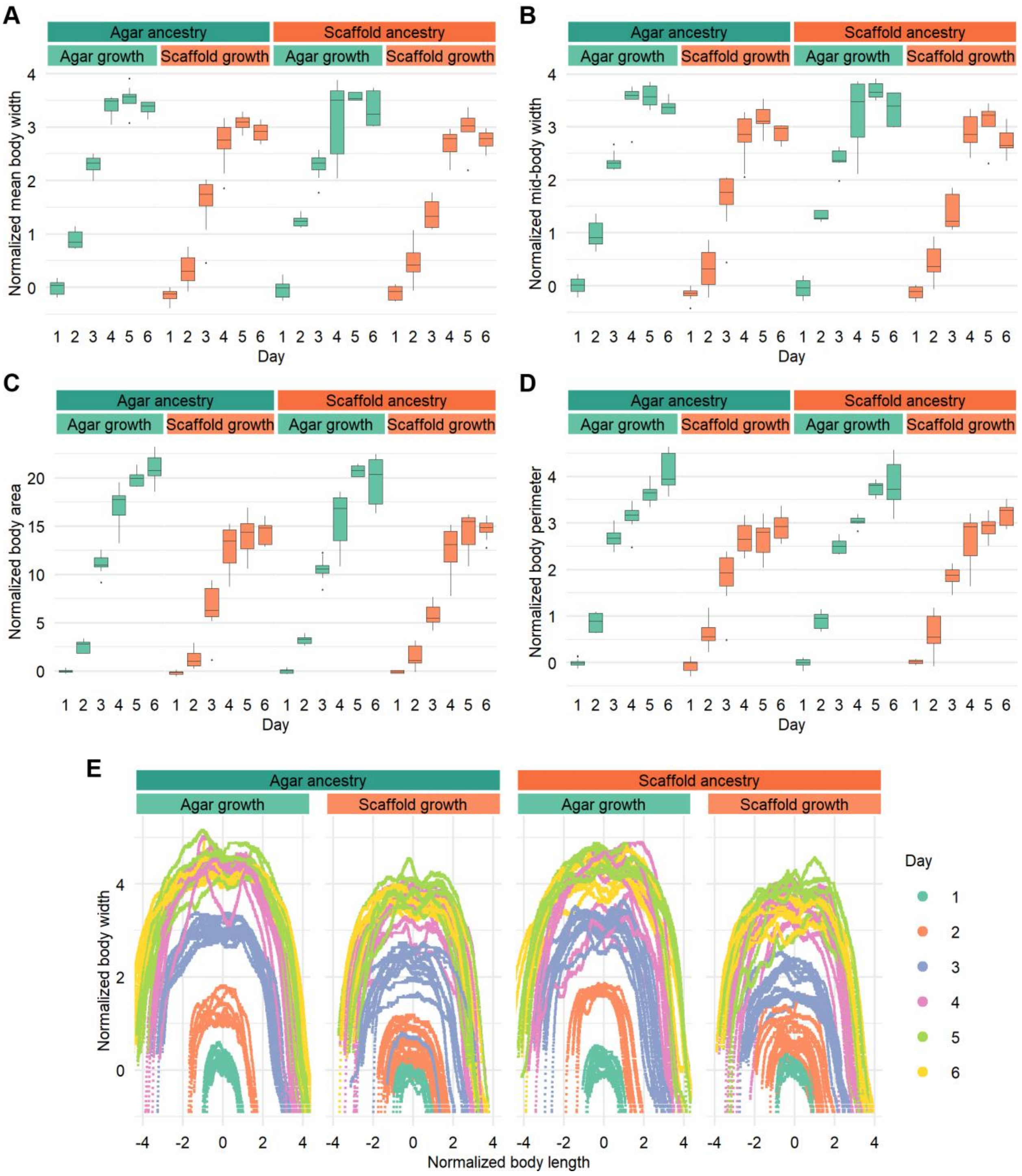

Fig. S2. Developmental dynamics of body morphology. (A) Mean body width, (B) mid-body width, (C) body area, and (D) body perimeter by day. (E) Body width along body length with mid-body centered at 0. Colors represent days post-hatching. All measured values are normalized to the mean of agar:agar worms on day 1. Sample sizes: same as Figs 2B–D.

Biology Open • Supplementary information

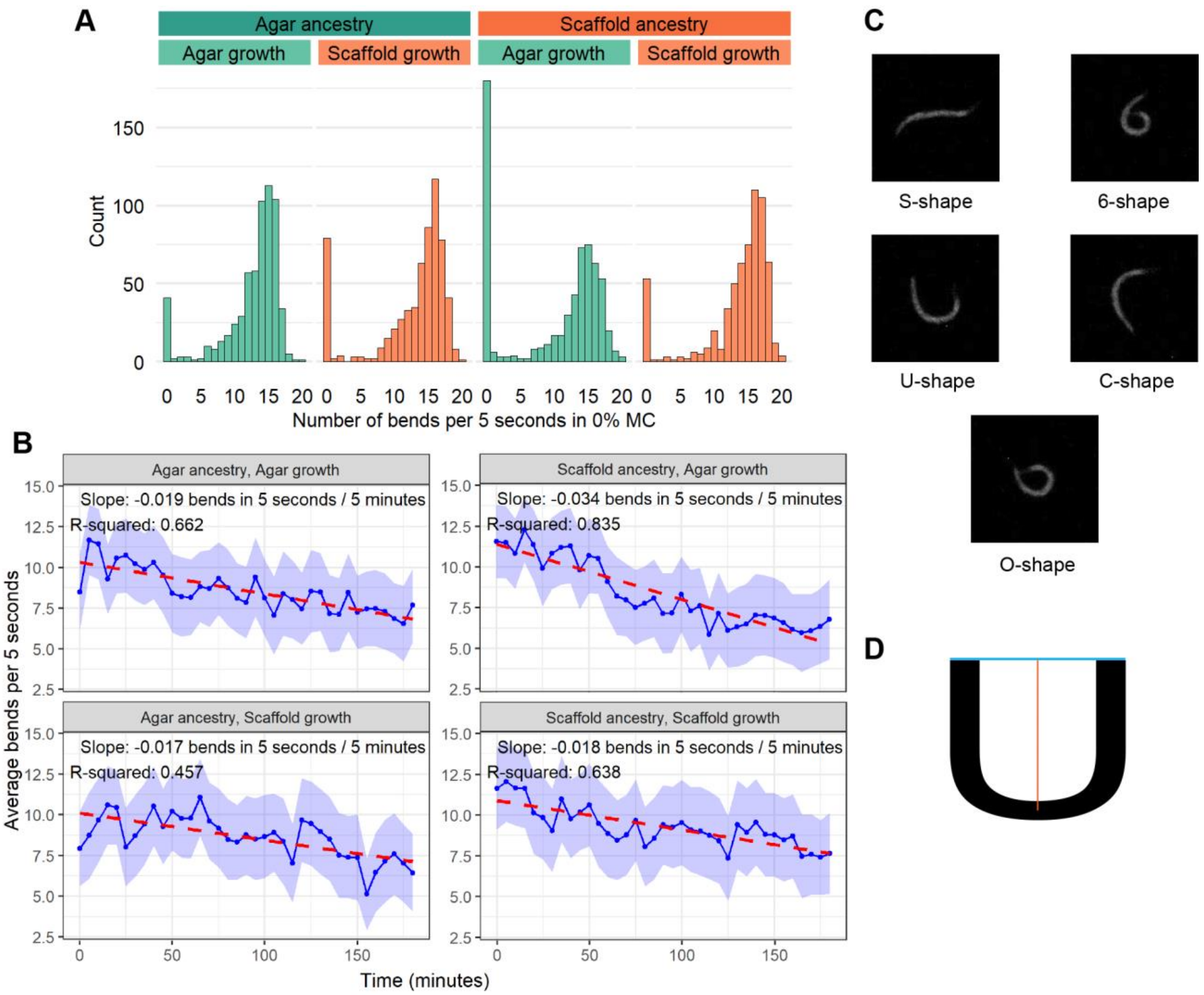

Fig. S3. Long-term swimming patterns reveal shifts in activity. (A) Histogram of number of swimming bends per 5 seconds in 0% MC. (B) Average number of bends per 5 seconds for each time point (point and line) with 95% confidence interval (ribbon) and linear regression (dashed line), in 0% MC. Text insets: slope and R-squared values. Sample sizes: same as Figs 3A–D. (C) Representative images of the 5 extracted body shapes: S, 6, U, C and O-shape. (D) Body amplitude measurement. A line (blue) is traced between the two ends of the worm spline (black), and the maximum bending amplitude is selected as the largest perpendicular distance (orange) between the end-to-end line and the worm spline.

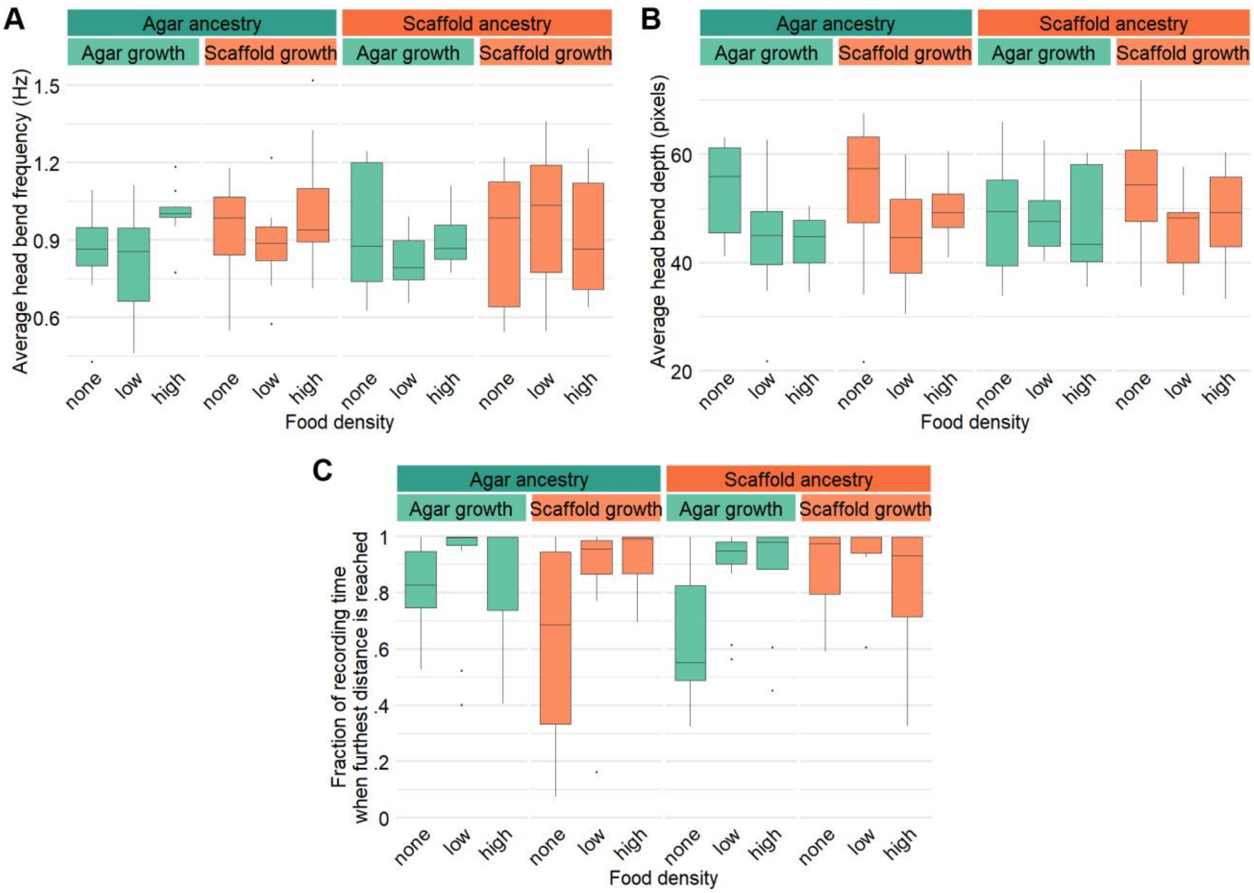

Fig. S4. Altered temporal exploration patterns during crawling. For one minute recordings. (A) Average head bend frequency (Hz) by food density. (B) Average head bend depth in pixels by food density. (C) Fraction of the recording period when the furthest Euclidean distance from the start point was reached by food density. Sample sizes: same as Fig. 6.

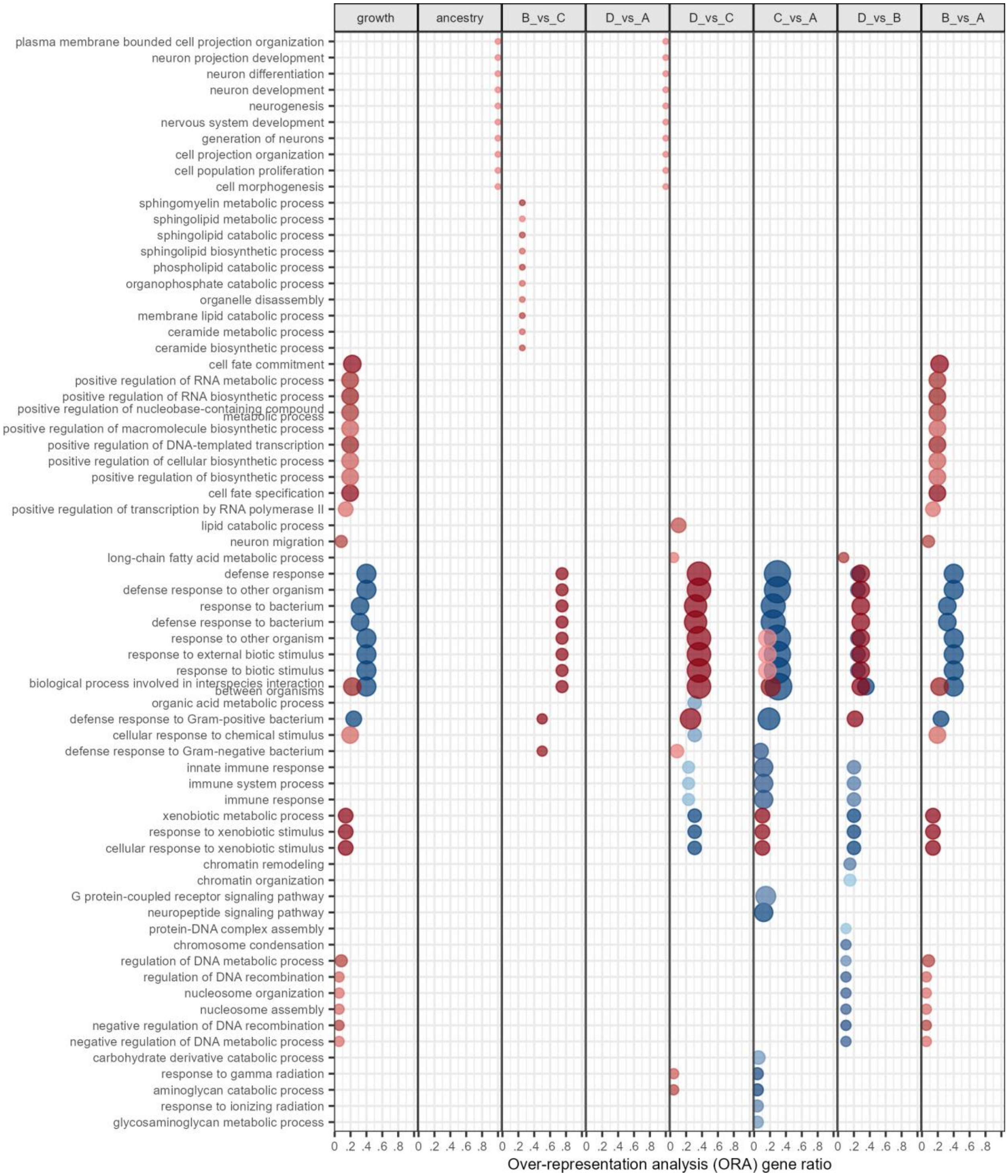

Fig. S5. Detailed over-representation analysis of Biological Process GO terms. Same legend as Fig. 8. Facet labels correspond to: “growth”: scaffold versus agar growth factor; “ancestry”: scaffold versus agar ancestry factor; “A”: agar:agar condition; “B”: agar:scaffold condition; “C”: scaffold:scaffold condition; “D”: scaffold:agar condition.

Biology Open • Supplementary information

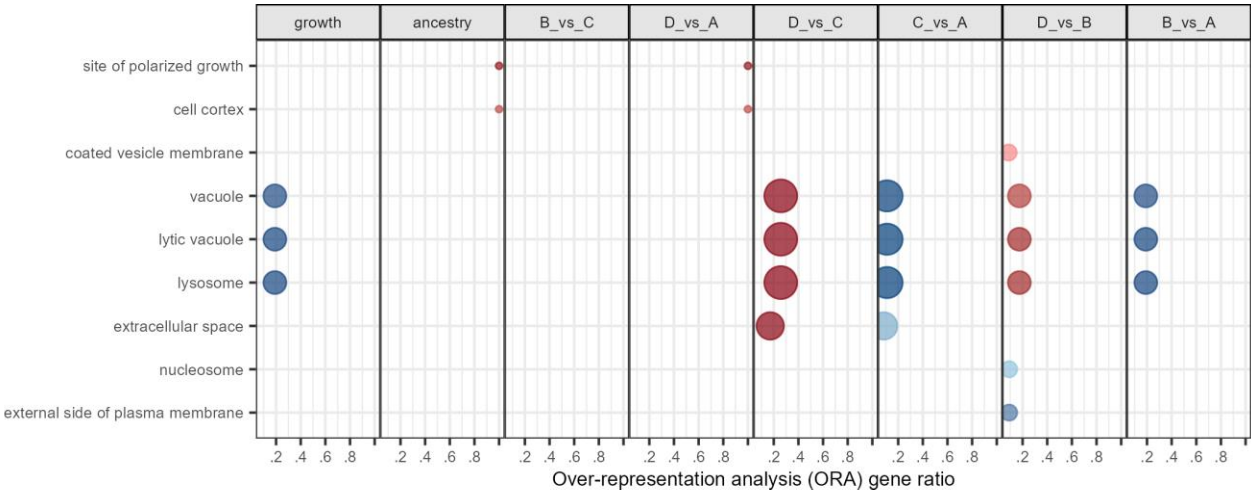

Fig. S6. Detailed over-representation analysis of Cellular Component GO terms. Same legend as Fig. 8. Same facet labels as Fig. S5.

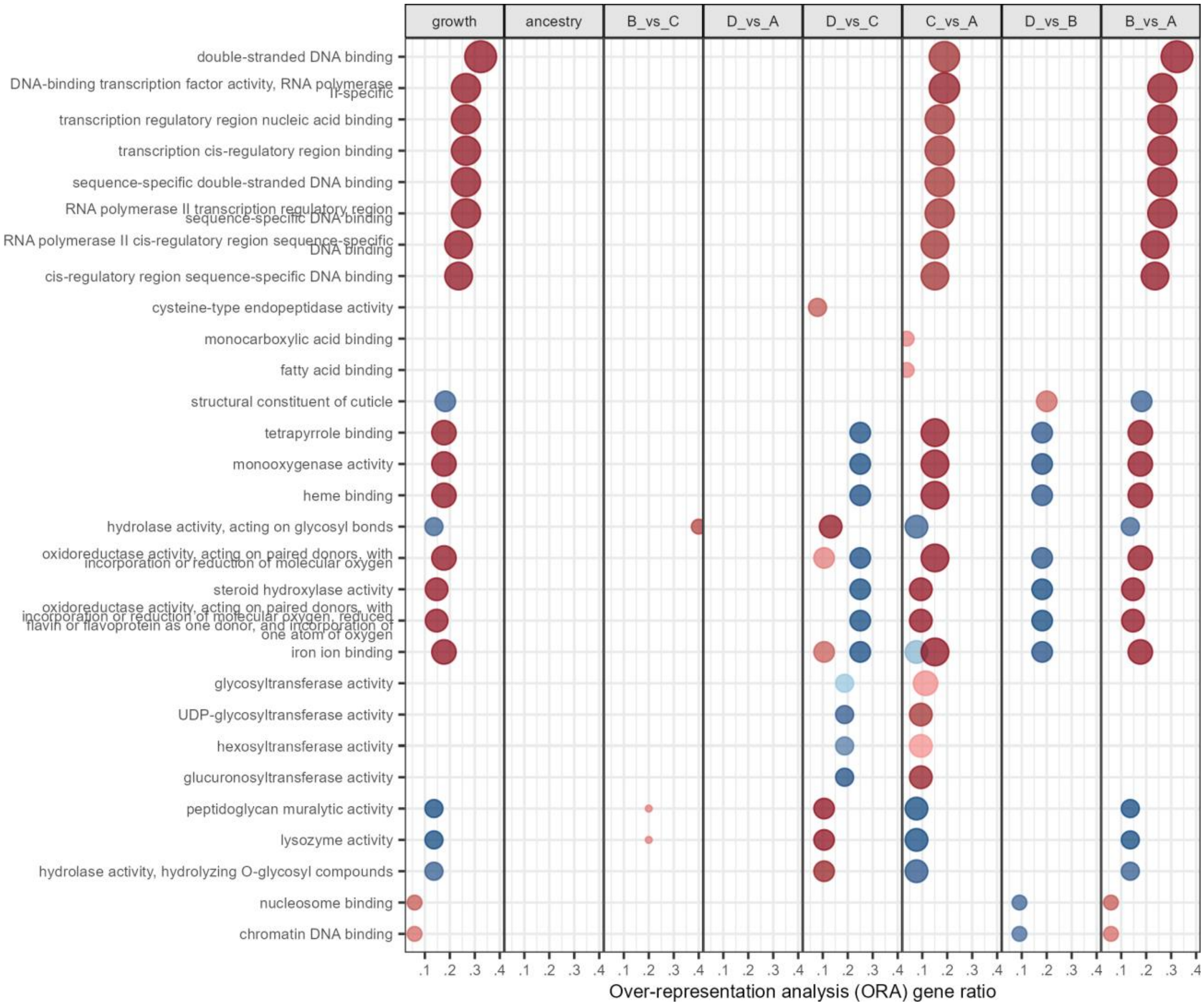

Fig. S7. Detailed over-representation analysis of Molecular Function GO terms. Same legend as Fig. 8. Same facet labels as Fig. S5.

Biology Open • Supplementary information

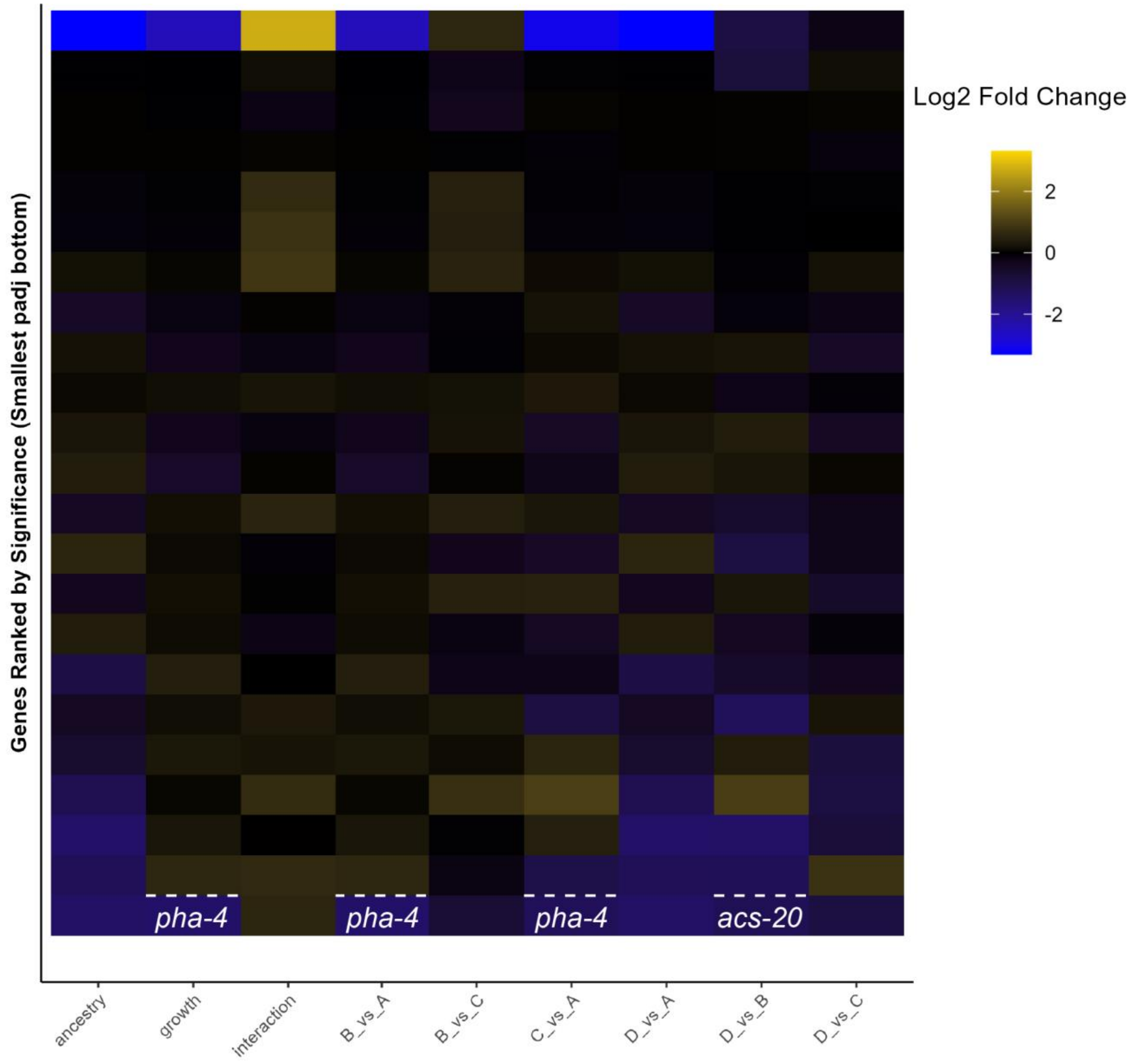

Fig. S8. Expression profile of genes commonly associated with calorie-restriction in *C. elegans*. Log_2fold change value of 25 genes commonly associated with calorie-restriction (*lgg-1, col-146, flp-21, acs-20, ragc-1, sbp-1, drr-2, T24B8.5, pha-4, aak-2, skn-1, eat-2, daf-2, clk-1, clk-2, clk-3, gro-1, zip-2, atf-7, nhr-62, ptr-8, nhr-23, sfa-1, sir-2.1, atx-2*) ordered by descending p-adjusted value. Dashed lines represent p-adj < 0.05. Absence of dashed line means no gene reached the significance threshold. Same facet labels as Fig. S5.

Biology Open • Supplementary information
